# Validity of the ithlete™ Smart Phone Application for Determining Ultra-Short-Term Heart Rate Variability

**DOI:** 10.2478/hukin-2013-0071

**Published:** 2013-12-31

**Authors:** Andrew A. Flatt, Michael R. Esco

**Affiliations:** 1 Department of Physical Education & Exercise Science, Human Performance Laboratory, Auburn University at Montgomery, USA.

**Keywords:** HrV, Athlete Monitoring, Parasympathetic, Mobile Device

## Abstract

The purpose of this investigation was to cross-validate the ithlete™ heart rate variability smart phone application with an electrocardiograph for determining ultra-short-term root mean square of successive R-R intervals. The root mean square of successive R-R intervals was simultaneously determined via electrocardiograph and ithlete™ at rest in twenty five healthy participants. There were no significant differences between the electrocardiograph and ithlete™ derived root mean square of successive R-R interval values (p > 0.05) and the correlation was near perfect (r = 0.99, p < 0.001). In addition, the ithlete™ revealed a Standard Error of the Estimate of 1.47 and Bland Altman plot showed that the limits of agreement ranged from 2.57 below to 2.63 above the constant error of −0.03. In conclusion, the ithlete™ appeared to provide a suitably accurate measure of root mean square of successive R-R intervals when compared to the electrocardiograph measures obtained in the laboratory within the current sample of healthy adult participants. The current study lays groundwork for future research determining the efficacy of ithlete™ for reflecting athletic training status over a chronic conditioning period.

## Introduction

Athletes exposed to high training loads and demanding competition schedules are at risk of experiencing unintentional overreaching, illness and injury when sufficient recovery is unattained. Heart rate variability (HRV), a non-invasive marker of autonomic status extrapolated from successive R peaks obtained by an electrocardiograph (ECG), is emerging as a valuable training status biometric used to objectively measure stress levels in athletes. A growing body of evidence supports the utility of HRV’s efficacy in sports training for the purposes of guiding periodization (Hautala et al., 2009; Kiviniemi et al., 2007; Kiviniemi et al., 2010), assisting in the diagnosis of over-trained states (Baumert et al., 2006; Tian et al., 2012); predicting physical performance (Chalencon et al., 2012; Manzi et al., 2009); and reflecting recovery status and training load (Chen et al., 2011; Iellamo et al., 2002; Pichot et al., 2000; Sartor et al., 2013).

Traditional HRV recordings are often performed in specialized laboratories and involve considerable time demand (i.e., at least 5-minute recordings) and a qualified technician for interpretation. These requirements make HRV assessment within athletic field settings difficult. Thus, practical measures capable of providing interpretable HRV data quickly, easily and affordably are desired. At present, there are various HRV field tools commercially available, though few have been validated. For example, several heart rate monitors have been shown to provide accurate R-R interval data, such as the Polar S810 (Gamelin et al., 2006; Gamelin et al., 2008; Nunan et al., 2009; Porto and Junqueira, 2009; Vanderlei et al., 2008; Weippert et al., 2010), the Polar RS800 (Wallén et al., 2012), and the Suunto T6 (Weippert et al., 2010). Though heart rate monitors provide more practicality than traditional HRV measures, they still require manual exportation of the raw data to a personal computer for software analysis and interpretation by an informed individual.

More recently, smart phone HRV applications that are used in conjunction with a chest-borne heart rate transmitter and a wireless ECG receiver are growing in popularity due to their low cost and enhanced practicality. A popular smart phone application named ithlete™ (HRV Fit Ltd. Southampton, UK) allegedly provides a time domain HRV index, the root mean square of successive R-R intervals (RMSSD), from a 55 second measurement. This parameter is respiratory sinus arrhythmia mediated; tracking the high frequency changes in heart rate rhythm in response to respiration and is reflective of cardiovascular parasympathetic modulation (Massin et al., 1999). The RMSSD appears to be a sensitive marker to both physical and non-physical related stressors (Tian et al., 2012). When considered with other athletic performance variables, changes in RMSSD throughout training and competition periods may indicate quality of physical adaptation, such as over-fatigue or increases in fitness (Cipryan et al., 2010; Oliveira et al., 2012; Tian et al., 2012). Additionally, it has been suggested that the RMSSD may be the preferred parameter for longitudinal athlete monitoring due to its easy calculation and interpretation (Plews et al., 2013) as well as its reduced sensitivity to breathing frequency compared to spectral measures (Pentilla et al., 2001; Saboul et al., 2013). This is particularly important when considering that field tools such as ithlete™ are intended for athlete use with unsupervised data collection. The prospect of a smart phone HRV application is attractive to coaches due to it being user friendly, affordable and time efficient. Therefore, it has potential for being utilized in field settings as an HRV monitoring tool among sports teams.

Unfortunately, there are no available studies that have examined the accuracy of the ithlete™ receiver and chest-strap hardware for determining HRV. This research is warranted as the merit of any field parameter depends on validity statistics when compared to laboratory derived measures. Therefore, the purpose of this investigation was to cross-validate the ithlete™ HRV smart phone application with an ECG for determining ultra-short-term RMSSD. Technology for acquiring heart rate data in the field has advanced considerably over time. Therefore, it is reasonable to hypothesize that the smart phone application with a heart rate strap and an ECG receiver will accurately reflect laboratory-derived HRV measures.

## Material and Methods

### Participants

Twenty-five male (n = 17) and female (n = 8) college students from the University’s Exercise Science program volunteered for this study. Descriptive statistics for the participants are shown in Table 1. Acquisition of the data occurred between the hours of 8:00 am and 12:00 pm on week days in the Human Performance Laboratory. Volunteers were told to report to the lab in a fasted state and to avoid the consumption of stimulants (e.g. coffee). Only healthy individuals were included in this study. Those with a heart condition, illness or prescription medication were excluded. All subjects provided written informed consent after being given a detailed account of the investigation, potential risks and were told that they may withdraw their participation at any time. The experimental protocol was granted ethical approval by the associated University’s Institutional Review Board.

### Procedures

For the ithlete™, each subject was fitted with a heart rate transmitter and an elastic strap (Non-Coded Polar T-31, Polar Electro Oy, Kemple, Finland) fastened securely around the upper thorax at the level of the xiphoid process. All ithlete™ HRV data was collected with the ithlete™ ECG receiver and ithlete™ application on an iPad2 (Apple Inc, Cupertino, California, USA). Since the ithlete™ was tested against HRV derived from an electrocardiogram (ECG), surface electrodes were also placed on each subject in a lead II configuration. The electrodes were connected to a Biopac MP100 data acquisition system (Goletta, CA, USA), which was connected to a Dell PC. Acknowledge software (v 3.9, BIOPAC, Goletta, CA, USA) was used to collect real-time ECG.

After preparing each subject for ithlete™ and ECG, they were instructed to rest in a supine position on a comfortable examination table in a quiet, dimly lit room with temperature controlled for at approximately 20.5° Celsius with 59% humidity. The testing protocol was administered after each subject remained at rest for a 15 minute acclimation period. Though the ECG ran continuously during this period, no heart rate data was recorded.

After the acclimation period, the conduction surface of the heart rate transmitter was moistened in preparation for the ithlete™ test. Next, the ithlete™ ECG receiver was inserted into the iPad2 headphone slot and the ithlete™ application was initiated. The iPad2 was held by a researcher no higher than 8 inches over the subject’s chest with the screen in clear view for the subject. The researcher initiated the measurement and the subject followed the breathing cadence of the application (i.e., approximately 7.5 breaths·min^−1^) which was easily displayed in both animated and written form on the screen to inform the user when to inhale and exhale. The duration of the ithlete™ measurement was 55 seconds. When the ithlete™ measurement was complete, the resulting RMSSD value was displayed on the iPad2 screen and recorded for data analysis. During the ithlete™ test, a second researcher stood at the Dell PC to manually mark the ECG strip of the Acknowledge software at the commencement and completion of the 55 second recording. If any non-sinus beats were found on the strip, the reading was excluded from analysis (n = 0). The ithlete™ application does not provide the user with an ECG strip. According to the manufacturer however, the device is equipped with an irregular beat detection and correcting process (Wegerif, 2009).

The ECG recordings were converted to a tachogram, which plotted the successive R-R intervals on the y-axis and the number of beats within the ECG segment on the x-axis, via specialized HRV software (Nevrokard version11.0.2, Izola, Slovenia). From the tachogram, the RMSSD of the 55 second test period was calculated. According to the ithlete™ manufacturer, the application modifies the RMSSD by taking the natural log transformation and multiplying by twenty (lnRMSSD × 20) providing a more intuitive and interpretable figure for the user on a ∼100 point scale (Wegerif, 2009). Therefore, the criterion RMSSD derived from the ECG was corrected accordingly to match the ithlete™ manufacturer’s instructions (i.e., log transformed and multiplied by 20).

### Statistical analysis

Means and standard deviations were determined for the raw and ln transformed ECG derived RMSSD values. The ithlete™ and corrected criterion RMSSD recordings were compared with a paired samples T-test and Pearson product correlation. In addition, the constant error (CE) and the standard error of estimate (SEE) were calculated for the ithlete™. Bland-Altman plots were also formed to identify the limits of agreement for ithlete™ (Bland and Altman, 1986). A priori statistical significance was set at p < 0.05. All statistical analysis was completed using the SPSS version 16.0.

## Results

**Figure 1:**
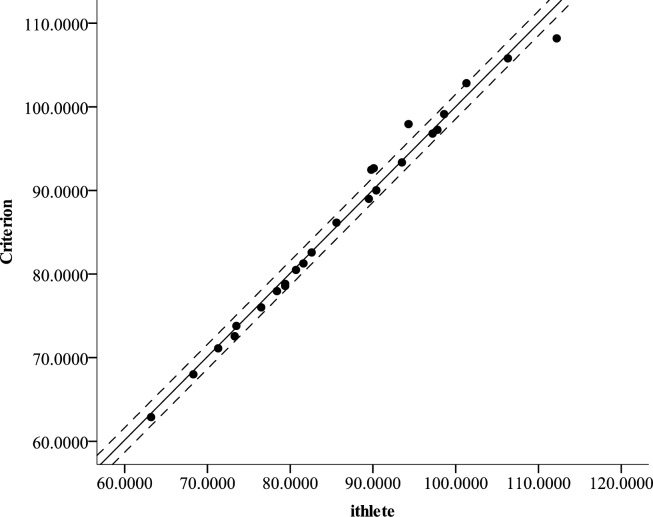
Scatterplot representing the relationship between the Criterion and ithlete™ The middle line represents the line of regression, while the two outside dashed lines represent the standard error of the estimate.

**Figure 2:**
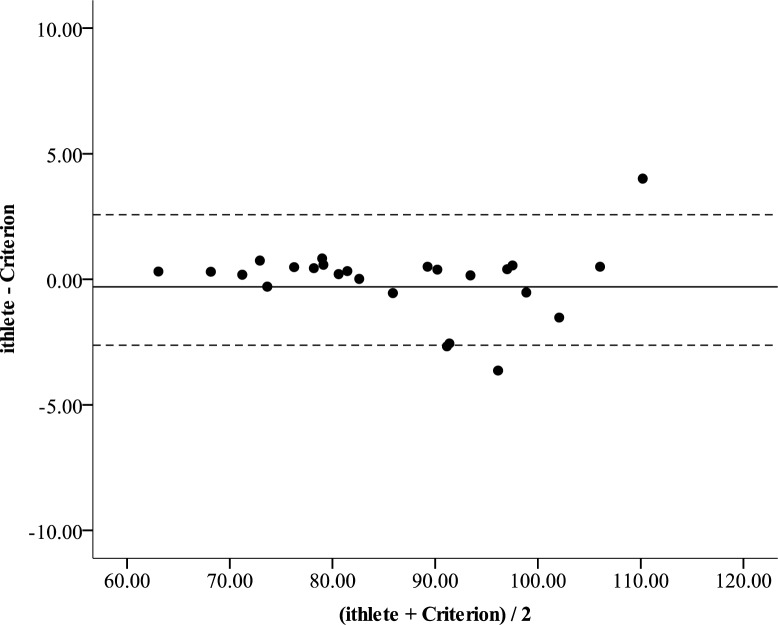
Bland-Altman plot comparing the corrected RMSSD estimated by the ithlete™ with the criterion. The solid line represents the mean bias while the two outside dashed lines represent the 95% limits of agreement.

The raw and ln transformed RMSSD values from the 55 sec ECG strip were 89.0 ± 54.2 ms and 4.3 ± 0.6, respectively. The corrected criterion RMSSD values were 86.23 ± 12.3, while the ithlete™ provided values of 86.19 ± 12.3. These values were not significantly different (p = 0.91) and the effect size was negligible (partial eta^2^ = 0.001). The correlation between the criterion and ithlete™ was near perfect (r = 0.99, p < 0.001, Figure 1). Compared to the criterion, the ithlete™ revealed a SEE of 1.47. The Bland Altman plot showed that the LOA ranged from 2.57 below to 2.63 above the CE of −0.03 (Figure 2).

## Discussion

The aim of this study was to determine the validity of the ithlete™ HRV smart phone application in comparison to laboratory derived RMSSD data. The mobile system functions via a wireless heart rate monitor, an analog chest strap and a portable ECG receiver that inserts into the headphone slot of a smart phone or tablet device. The heart rate monitor detects cardiac cycles at the moistened conduction site and sends this information via radio transmission to the receiver. The receiver processes R wave data and automatically performs a calculation providing the lnRMSSD multiplied by 20. The manufacturer purports that the ithlete™ is capable of providing accurate values (Wegerif, 2009), however, to our knowledge, there are no previous studies that have investigated these claims. The current study demonstrated that the ithlete™ system strongly agreed with the criterion measure. There were no significant mean differences and a near perfect correlation between the ithlete™ and laboratory corrected RMSSD values. In addition, the ithlete™ provided a low SEE and tight LOA. Therefore, the ithlete™ provided a suitably accurate measure of corrected RMSSD when compared to the ECG measure obtained in the laboratory within the current sample of healthy adult participants.

Previous studies comparing the accuracy of other portable HRV devices have primarily focused on wrist-worn heart rate monitors with chest strap transmitters, which provide several measures of HRV (e.g., RMSSD, standard deviation of normal-to-normal intervals [SDNN], low [LF] and high [HF] frequency spectral power). When cross-validated, the monitors appear to provide similar statistics compared to the findings of the ithlete™ in the current study (Gamelin et al., 2008; Gamelin et al., 2006; Nunan et al., 2009). For instance, strong correlations (r values ranging from 0.85 to 0.99) between the Polar S810 and laboratory derived HRV parameters have been previously reported in adults (Gamelin et al., 2006; Nunan et al., 2009; Weippert, 2010) and children (Gamelin et al., 2008). Weippert et al. (2010) showed excellent agreement between the Suunto t6 and an ambulatory ECG system for measuring resting HRV.

Studies comparing the accuracy of smart phone devices for providing HRV data are limited. To our knowledge, only one study has been made available. Recently, Heathers (2013) showed that a smart phone application capable of providing HRV data via pulsewave finger sensor and ECG receiver provided strong agreement to laboratory ECG measures (correlation values ranging from 0.965 to 0.997). However, the pulsewave finger sensor is not as commonly used as conventional chest straps within athletic field settings for heart rate data collection.

Breathing is paced by the ithlete™ system at a cadence of 7.5 breaths per minute during HRV recording. This breathing tempo reflects the mean spontaneous breathing frequency of a sample of healthy adult runners (Saboul et al., 2013). The manufacturer has elected paced breathing to add a component of reliability to the conditions of the measurement process when taken daily (Wegerif, 2009). This may only be precautionary, however, as it has been recently shown that breathing rate does not appear to influence RMSSD, unlike spectral indexes (Pentilla et al., 2001; Saboul et al., 2013). Collectively, these findings suggest that RMSSD is less sensitive to the respiratory rate compared to frequency domain parameters (e.g., HF and LF) thus making it more suitable for assessment in field settings under ambulatory resting conditions.

The ithlete™ provides only one measure of HRV, i.e., the RMSSD. This global marker of cardiovascular-parasympathetic modulation has been shown to be an important value with regard to athlete monitoring. Descending RMSSD during intensive training has been associated with increased fatigue (Baumert et al., 2006; Pichot et al., 2002; Thiel et al., 2011) and overtraining (Plews et al., 2012; Tian et al., 2012). Increases in RMSSD can be indicative of improvements in fitness when performance markers increase (Boullosa et al., 2012; Chalencon et al., 2012; Oliveira et al., 2012). Alternatively, when performance markers decrease, increases in RMSSD from baseline can also indicate high fatigue or overtraining (Le Meur et al., 2013; Tian et al., 2012). Therefore, it has been recommended that the HRV parameters such as RMSSD be considered with other markers of fatigue and performance for meaningful interpretation (Bosquet et al., 2008). Since the ithlete™ appears to accurately provide a measure of RMSSD, it has potential for use as a training status metric in athletic field settings.

In addition, the ithlete™ provides a measure of RMSSD in a relatively short time period of only 55 seconds. This could be considered a limitation of the device as recordings of at least 5-minutes in duration have typically been recommended to establish short-term HRV measurements (Task Force, 1996). However, previous investigations in healthy and clinical populations have shown excellent agreement when comparing ultra-short-term RMSSD measures of equal to or less than 60-seconds to criterion recordings of 5-minutes (Nussinovitch et al., 2012; Nussinovitch et al., 2011; Thong et al., 2003). Salahuddin et al. (2007) found the RMSSD to be a reliable measure when assessed in as low as 10 second measures for monitoring mental stress in mobile settings. It has also been shown that 10 second RMSSD measurements are capable of reflecting cardiac vagal tone in comparison to standard 5 minute measures (Hamilton et al., 2004). In addition, ultra-short term measures enhance convenience and practicality verses longer duration measurements (Katz et al., 1999), which could potentially enhance compliance and increase the likelihood of usage among sports teams.

The present study is not without possible limitations. First, we did not perform repeated trials to test reliability. However, HRV itself is not a reproducible physiological marker, as drastic differences between test-retest trials have been reported (Cipryan and Litschmannova, 2013; Sandercock et al., 2005). Second, each measurement was carefully performed by researchers to ensure measurement quality, not by athletes in the field. Third, measures were only tested in a supine position in accordance with standardized methods (Task Force, 1996). Seated or standing measurements may be preferred for athletes (Kiviniemi et al., 2007) and therefore testing ithlete™ accuracy in these postures requires validation. Last, the group of subjects was not analyzed across a chronic training period. Evidently, future research should aim to determine if the ithlete™ can reflect fatigue or performance changes throughout a conditioning program. The novel findings of the current study will be important with longitudinal follow-up of athletes throughout training or competition periods.

In conclusion, the current study showed that under controlled, laboratory conditions, the ithlete™ values mirrored ECG derived and corrected measures of RMSSD in healthy adult subjects. These findings lend initial support to the prospective application of smart phone derived HRV tools intended for non-expert use (e.g. athletes) in field settings. Acquiring accurate HRV data conveniently with a smart phone and the ithlete™ system for the purposes of athlete monitoring appears promising.

## Figures and Tables

**Table 1:** Descriptive statistics (mean ± SD) of the studied sample

	Men (n = 17)	Women (n = 8)	All (n = 25)
Age (yr)	26.7 ± 4.2	25.4 ± 4.6	26.3 ± 4.2
Height	180.6 ± 5.7^*^	162.1 ± 5.8	175.3 ± 11.2
BM (kg)	91.2 ± 18.3^*^	61.6 ± 9.4	82.7 ± 21.0
BMI	27.7 ± 4.9^*^	23.0 ± 2.4	25.7 ± 4.6

* Significantly higher than women (p < 0.05).
